# Dietary factors and hypertension: A Mendelian randomization analysis

**DOI:** 10.1002/fsn3.3931

**Published:** 2024-01-28

**Authors:** Fanfan Li, Wenwen Yang, Shuo Sun, Wenhui He, Shangqing Xu, Biao Han, Minjie Ma

**Affiliations:** ^1^ Department of Thoracic Surgery The First Hospital of Lanzhou University Lanzhou China; ^2^ Gansu University of Chinese Medicine Lanzhou China; ^3^ The First Clinical Medical College Lanzhou University Lanzhou China; ^4^ Skills Training Center The First Clinical Medical College of Lanzhou University Lanzhou China; ^5^ Gansu Province International Cooperation Base for Research and Application of Key technology of Thoracic Surgery Lanzhou China

**Keywords:** dietary consumption, hypertension, Mendelian randomization

## Abstract

This research explores the causal link between dietary habits and hypertension through Mendelian randomization, providing distinct perspectives on the role of diet in addressing this worldwide health issue. Utilizing instrumental variables, we applied advanced statistical methods, including the weighted median, inverse variance weighted, and MR‐Egger, to evaluate the impact of 17 dietary elements on hypertension. These elements ranged across various food groups, such as fruits, meats, vegetables, and beverages, both alcoholic and nonalcoholic. Our results identified a significant positive association of hypertension with weekly alcohol consumption (OR 1.340 [95%CI 1.0001 to 1.794], *p* = .0499) and poultry intake (OR 2.569 [95%CI 1.305 to 5.057], *p* = .00631). Conversely, a negative association was observed with lamb/mutton (OR 0.550 [95%CI 0.343 to 0.881], *p* = .0129), cheese (OR 0.650 [95%CI 0.519 to 0.813], *p* = .000159), tea (OR 0.797 [95%CI 0.640 to 0.993], *p* = .0433), cereal (OR 0.684 [95%CI 0.494 to 0.948], *p* = .0227), and dried fruit consumption (OR 0.492 [95%CI 0.343 to 0.707], *p* = .000127). These findings suggest that dietary modifications, such as increasing consumption of specific foods like cheese, lamb/mutton, tea, cereals, and dried fruits, could potentially reduce hypertension risk while reducing intake of alcoholic beverages and poultry might mitigate its increase. No direct causal relationships were established between other dietary factors and hypertension. The study highlights the importance of specific dietary modifications for the prevention and control of hypertension, making a substantial contribution to public health tactics and recommendations.

## Graphical abstract

This study utilizes Mendelian randomization to explore the relationship between dietary factors and hypertension. Key findings indicate a positive association of hypertension with alcohol and poultry consumption, and a negative association with lamb/mutton, cheese, tea, cereal, and dried fruit consumption. These results suggest potential dietary modifications for managing hypertension, highlighting the significance of targeted dietary changes in public health strategies.

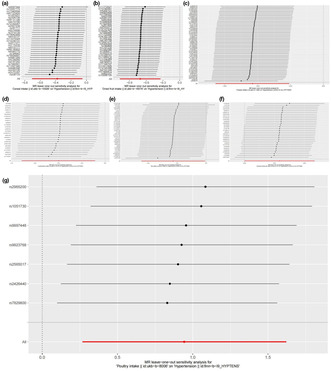

## 1 INTRODUCTION

Hypertension is a global issue that endangers people's health (Zhou et al., 2021). Elevated blood pressure levels can result from heightened cardiac output, increased peripheral vascular resistance, or a combination of both, adversely affecting the average lifespan (Kitt et al., 2019). Familial clustering implies a genetic susceptibility whose interaction with environmental factors (such as salt and calorie consumption) ultimately determines the severity of the rise in blood pressure. Nearly half of the population is endangered by potential hypertension (Williams et al., 2018a). The danger of hypertension is mainly due to heart disease, chronic kidney disease, and cardiovascular and cerebrovascular pathologies (Guo et al., 2020). Understanding the impact of particular diets on blood pressure readings is crucial from a clinical perspective (Saklayen, 2018). The first thing that people with hypertension take is nonmedication to control their blood pressure, and even after taking medication, they should also maintain good dietary habits. The most important nonpharmaceutical treatment measures are a low‐salt diet, adequate potassium consumption, and a healthy balanced diet (Williams et al., 2018b). An earlier study found that the DASH diet lowered blood pressure (Sacks et al., 2001), numerous dietary recommendations, such as reducing sodium intake, consuming low‐fat dairy products, limiting sweets and red meat, and increasing fruit and vegetable consumption, have been shown to effectively lower blood pressure in recent years. Numerous research studies have demonstrated that high carbohydrate consumption appears to exacerbate metabolic syndrome (MS; Taskinen et al., 2019), conversely, carbohydrate‐restricted dietary regimens appear to be more effective in controlling the characteristics of MS (Volek et al., 2009). Increasing the intake of fresh fruits and vegetables, rich in potassium, antioxidants, and flavonoids, while reducing dietary sodium, can effectively lower blood pressure across the population (Borgi et al., 2016). People's health comes from changes in long‐term dietary habits (Sacks et al., 2001). Advocating for a healthy DASH eating plan can impact the diverse physiological processes regulating blood pressure, thereby diminishing the risk of cardiovascular disease (Filippou et al., 2022).

Observational research has consistently demonstrated a clear connection between dietary patterns and the prevalence of hypertension (Tseng et al., 2021). Mendelian randomization (MR) uses instrumental variable (IV) consisting of genetic variation and has advantages compared to other study methods. Mendelian randomization (MR) studies investigating the cause‐and‐effect relationship between dietary factors and hypertension have been relatively scarce. Thus, our research focuses on exploring the causal connection between diet and hypertension through Mendelian randomization.

## 2 METHODOLOGY

In Mendelian randomization analysis, choosing appropriate instrumental variables is crucial. These variables should be strongly associated with exposure factors but not influenced by confounding or outcome factors. The primary data for our study were processed by the MRC Integrative Epidemiology Unit (IEU) at the University of Bristol, utilizing information from the UK Biobank and the FinnGen Biobank, as of 2018. GWAS data (https://gwas.mrcieu.ac.uk/) are public, anonymous, and deidentified.

### 2.1 Data sources and the selection of IVs

We obtained summary data from genome‐wide association studies (GWAS) of 17 dietary patterns, sourced from the UK Biobank. This expansive prospective cohort encompasses around 500,000 individuals, providing both genetic and a range of phenotypic details (Sudlow et al., 2015). The dietary factors under consideration include consumption of fresh fruits, pork, cooked vegetables, poultry, beef, dried fruits, nonoily fish, alongside frequency of alcohol consumption. Also included are oily fish, lamb/mutton, bread, cereals, salads/raw vegetables, weekly intake of alcoholic drinks, as well as consumption of tea, coffee, and cheese. This study aims to explore the cause‐and‐effect relationships among 17 different dietary factors and hypertension. All data for hypertension were extracted from the FinnGen Biobank. The FinnGen biobank contains sufficient SNPs (SNPs > 10^7^ in the hypertension dataset), so no proxy SNPs were used in this study (gwas.mrcieu.ac.uk). SNPs were at clustering window >10,000 kb, the genome‐wide significance level (*p* < 5 × 10^−8^), and linkage disequilibrium level (*r* ^2^ < .001; Table 1). In the Mendelian randomization analysis, exposure factors were vicariously established with the help of instrumental variables to achieve a relationship with the outcome variable. IVs consist of multiple SNPs. SNPs used for each dietary factor ranged from 5 and 90 (Table 2). *F* > 10 indicates a strong association between the representative instrumental variable and the exposure factor (Staiger & Stock, 1994). The *F*‐statistics in this study were all >10 (range: 17.571 to 116.369).

**TABLE 1:** Information of the exposures and outcome datasets.

IEU GWAS id	Exposure or outcome	Identified SNPs	Participants included in the analysis	*F*‐statistic
ieu‐b‐73	Alcoholic drinks per week	31	335,394 European‐descent individuals	102.432
ukb‐b‐5779	Alcohol intake frequency	89	462,346 European‐descent individuals	116.369
ukb‐b‐8006	Poultry intake	7	461,900 European‐descent individuals	24.411
ukb‐b‐2862	Beef intake	10	461,053 European‐descent individuals	26.644
ukb‐b‐17,627	Nonoily fish intake	9	460,880 European‐descent individuals	23.114
ukb‐b‐2209	Oily fish intake	58	460,443 European‐descent individuals	37.251
ukb‐b‐5640	Pork intake	13	460,162 European‐descent individuals	18.071
ukb‐b‐14,179	Lamb/mutton intake	26	460,006 European‐descent individuals	18.023
ukb‐b‐11,348	Bread intake	25	452,236 European‐descent individuals	38.339
ukb‐b‐1489	Cheese intake	58	451,486 European‐descent individuals	45.070
ukb‐b‐8089	Cooked vegetable intake	16	448,651 European‐descent individuals	20.679
ukb‐b‐6066	Tea intake	37	447,485 European‐descent individuals	64.142
ukb‐b‐3881	Fresh fruit intake	50	446,462 European‐descent individuals	15.716
ukb‐b‐15,926	Cereal intake	33	441,640 European‐descent individuals	33.848
ukb‐b‐1996	Salad/raw vegetable intake	18	435,435 European‐descent individuals	17.571
ukb‐b‐5237	Coffee intake	35	428,860 European‐descent individuals	31.703
ukb‐b‐16,576	Dried fruit intake	38	421,764 European‐descent individuals	25.348
finn‐b‐I9_HYPTENS	Hypertension	N/A	55,917 European‐descent cases and 162,837 European‐descent controls	N/A

*Note*: The information of the exposure and outcome datasets.

Abbreviations: GWAS, Genome‐Wide Association Studies; c, Integrative Epidemiology Unit; SNPs, single‐nucleotide polymorphisms.

**TABLE 2:** The results of Mendelian randomization analyses.

		Exposure	Used SNPs	Inverse variance weighted method (Wald ratio)		Weighted median method		MR‐Egger method		Cochrane's Q test		Pleiotropy			MR‐PRESSO^a^							Outliers excluded										
				OR (95% CI)	*p*‐value	OR (95% CI)	*p*‐value	OR (95% CI)	*p*‐value	Q	*p*‐value	MR‐Egger intercept	SE	*p*‐value	Raw			Outliesrs	Outlier‐corrected			Inverse variance weighted method		Weighted median method		MR‐Egger method		Cochrane's Q test		Pleiotropy		
															Casual estimate	SD	*p*‐value		Casual estimate	SD	*p*‐value	OR (95% CI)	*p*‐value	OR (95% CI)	*p*‐value	OR (95% CI)	*p*‐value	Q	*p*‐value	MR‐Egger intercept	SE	*p*‐value
1	ieu‐b‐73	Alcoholic drinks per week	33	1.177 (0.833–1.663)	.356	1.493 (1.040–2.143)	.030	2.085 (0.967–4.494)	.0703	76.042	<.0001	−0.0109	0.00668	.115	0.163	0.176	.363	rs10085696;rs331939	0.292	0.149	.0592	1.340 (1.0001–1.794)	.0499	1.514 (1.044–2.195)	.0288	1.958 (1.026–3.737)	.0508	48.675	.0170	−0.00732	0.00569	.208
2	ukb‐b‐5779	Alcohol intake frequency	92	1.181 (1.020–1.367)	.0259	1.119 (0.947–1.323)	.187	0.922 (0.587–1.448)	.725	233.846	<.0001	0.00621	0.00547	.259	0.166	0.0746	.0284	rs1421085;rs34811474;rs73050128	0.0966	0.0639	.135	1.101 (0.972–1.248)	.131	1.103 (0.935–1.302)	.245	0.941 (0.641–1.379)	.754	160.872	<.0001	0.00398	0.00465	.394
3	ukb‐b‐8006	Poultry intake	7	2.569 (1.305–5.057)	.00631	2.795 (1.191–6.562)	.0182	325,129.5 (0.000472–2.239e+14)	.276	2.668	.849	−0.127	0.112	.309	0.944	0.230	.00639	NA	NA	NA	NA	NA	NA	NA	NA	NA	NA	NA	NA	NA	NA	NA
4	ukb‐b‐2862	Beef intake	14	0.926 (0.303–2.836)	.893	0.798 (0.351–1.815)	.591	0.501 (0.000455–551.343)	.850	82.544	<.0001	0.00781	0.0448	.864	−0.0765	0.571	.895	rs132901;rs1421085;rs1470610;rs62396185	0.213	0.375	.584	1.238 (0.593–2.581)	.570	0.784 (0.328–1.875)	.584	0.171 (0.00320–9.109)	.409	15.822	.0707	0.0244	0.0246	.350
5	ukb‐b‐17,627	Nonoily fish intake	11	1.825 (0.609–5.467)	.283	0.958 (0.426–2.155)	.917	0.798 (0.00328–194.228)	.937	46.078	<.0001	0.0103	0.0340	.770	0.601	0.560	.308	rs838133;rs56094641	0.308	0.313	.354	1.360 (0.730–2.533)	.332	1.053 (0.452–2.453)	.904	2.156 (0.0805–57.692)	.661	7.774	.456	−0.00534	0.0190	.787
6	ukb‐b‐2209	Oily fish intake	60	0.854 (0.630–1.159)	.312	0.749 (0.547–1.026)	.0722	0.680 (0.187–2.472)	.560	158.710	<.0001	0.00340	0.00953	.722	−0.157	0.155	.316	rs1421085;rs9606833	−0.232	0.132	.0843	0.793 (0.612–1.027)	.0789	0.742(0.542–1.016)	.0628	0.459 (0.156–1.356)	.164	104.026	.000143	0.00806	0.00791	.313
7	ukb‐b‐5640	Pork intake	13	1.948 (0.907–4.183)	.0873	1.478 (0.649–3.365)	.353	1.267 (0.00874–183.611)	.928	23.545	.0234	0.00447	0.0260	.867	0.667	0.390	.113	NA	NA	NA	NA	NA	NA	NA	NA	NA	NA	NA	NA	NA	NA	NA
8	ukb‐b‐14,179	Lamb/mutton intake	30	0.879 (0.457–1.690)	.698	0.432 (0.232–0.804)	.00815	0.957 (0.0581–15.767)	.976	93.887	<.0001	−0.00095	0.0154	.951	−0.129	0.334	.701	rs1556147;rs276453;rs62106258;rs429358	−0.598	0.240	.0200	0.550 (0.343–0.881)	.0129	0.389 (0.219–0.689)	.00120	0.195 (0.0171–2.222)	.200	33.461	.120	0.0108	0.0127	.403
9	ukb‐b‐11,348	Bread intake	25	0.775 (0.521–1.153)	.208	0.929 (0.592–1.457)	.748	0.688 (0.105–4.487)	.699	48.208	.00238	0.00173	0.0136	.900	−0.255	0.203	.220	NA	NA	NA	NA	NA	NA	NA	NA	NA	NA	NA	NA	NA	NA	NA
10	ukb‐b‐1489	Cheese intake	60	0.663 (0.517–0.850)	.00117	0.675 (0.514–0.885)	.00443	1.945 (0.698–5.419)	.208	130.804	<.0001	−0.0186	0.00879	.0385	−0.411	0.127	.00193	rs10938397;rs919109	−0.431	0.114	.000381	0.650 (0.519–0.813)	.000159	0.673 (0.510–0.888)	.00506	1.409 (0.553–3.593)	.476	99.325	.000441	−0.0134	0.00804	.101
11	ukb‐b‐8089	Cooked vegetable intake	17	1.480 (0.615–3.561)	.381	1.372 (0.703–2.679)	.354	2.159 (0.000102–45,688.468)	.881	58.324	<.0001	−0.00390	0.0523	.942	0.392	0.448	.394	rs1421085	0.00792	0.297	.979	1.008 (0.563–1.803)	.979	1.291 (0.650–2.567)	.466	1.446 (0.00245–852.779)	.911	22.328	.0995	−0.00373	0.0335	.913
12	ukb‐b‐6066	Tea intake	39	0.770 (0.577–1.027)	.0754	0.777 (0.585–1.033)	.0825	1.231 (0.666–2.277)	.511	103.354	<.0001	−0.0101	0.00599	.101	−0.261	0.147	.0834	rs57462170;rs9937354	−0.226	0.112	.0508	0.797 (0.640–0.993)	.0433	0.776 (0.577–1.044)	.0933	1.125 (0.708–1.785)	.621	54.989	.0223	−0.00747	0.00453	.108
13	ukb‐b‐3881	Fresh fruit intake	52	0.972 (0.630–1.497)	.896	1.147 (0.694–1.896)	.5930	3.075 (0.723–13.083)	.135	103.747	<.0001	−0.0111	0.00679	.109	−0.0288	0.221	.897	rs12780952;rs9919429	0.117	0.189	.538	1.124 (0.777–1.628)	.535	1.153 (0.680–1.956)	.597	2.086 (0.595–7.311)	.256	71.003	.0216	−0.00600	0.00594	.317
14	ukb‐b‐15,926	Cereal intake	38	0.793 (0.492–1.279)	.343	0.622 (0.420–0.921)	.0178	0.926 (0.117–7.302)	.942	149.086	<.0001	−0.00226	0.0150	.881	−0.231	0.243	.349	rs10857964;rs11038810;rs2799849;rs4988235;rs62442924	−0.379	0.167	.0295	0.684 (0.494–0.948)	.0227	0.602 (0.402–0.903)	.0142	0.932 (0.238–3.649)	.920	53.319	.0104	−0.00463	0.0102	.651
15	ukb‐b‐1996	Salad/raw vegetable intake	18	0.795 (0.453–1.397)	.426	0.951 (0.441–2.051)	.897	2.216 (0.160–30.688)	.561	19.710	.289	−0.0111	0.0142	.445	−0.228	0.287	.437	NA	NA	NA	NA	NA	NA	NA	NA	NA	NA	NA	NA	NA	NA	NA
16	ukb‐b‐5237	Coffee intake	38	1.265 (0.873–1.832)	.214	0.811 (0.557–1.182)	.276	1.011 (0.477–2.144)	.977	113.471	<.0001	0.00422	0.00628	.506	0.2350	0.189	.222	rs1421085;rs2472297;rs476828	0.170	0.171	.328	1.185 (0.847–1.658)	.321	1.490 (1.007–2.206)	.0460	1.275 (0.577–2.815)	.552	60.231	.00366	−0.00116	0.00581	.843
17	ukb‐b‐16,576	Dried fruit intake	39	0.531 (0.355–0.794)	.00204	0.386 (0.255–0.585)	<.0001	0.129 (0.0226–0.735)	.0268	90.638	<.0001	0.0177	0.0108	.110	−0.632	0.205	.00379	rs17175518	−0.709	0.185	.000476	0.492 (0.343–0.707)	.000127	0.379 (0.250–0.575)	<.0001	0.135 (0.0285–0.640)	.0162	70.766	.000689	0.0162	0.0097	.103

Abbreviations: CI, confidence interval; NA, not available; OR, odds ratio; SNPs, single‐nucleotide polymorphisms.

^a^ The results of MR‐PRESSO are presented in the form of beta values, and there is a conversion relationship between beta values and OR, specifically beta = log(OR).

^b^ We repeated the Mendelian randomization analysis after removing outliers.

In conducting this Mendelian randomization (MR) analysis, we exclusively analyzed anonymized databases, thereby ensuring no impact on patients. The study utilized GWAS summary statistics from published studies and publicly accessible summary data available online. Each GWAS included had previously secured ethical approval. The summary statistics used were deidentified, readily downloadable, and available for unrestricted use.

### 2.2 Statistical analysis

IVW was used for the calculation of causal effects and had the highest utility among all methods for calculating two‐sample analysis (Hartwig et al., 2017). The MR‐Egger method allows all invalid IVs and the weighted median allows 50% or less of invalid IVs. The results are more accurate when all methods show a correlation. Cochran's Q test was used to test the heterogeneity when *p* < .05; however, the presence of heterogeneity has little to do with the accuracy of the model. MR‐Egger was used to measure directional multilateralism and allowed for the presence of nonzero intercepts. Changes in results after SNP removal were analyzed by the leave‐one‐out method. Outliers are removed as soon as they are discovered by MR‐PRESSO. Then, the MR analysis reran using the TwoSampleMR package in R software version (4.2.0; Hemani et al., 2018).

## 3 RESULTS

Dietary factors were collected in 335,394 to 462,346 European individuals. The results included 20,629 cases of hypertension in European individuals and 135,449 controls of European descent, with little overlap between exposure and the populations involved in the results. Table S1 provides additional information on outcome and exposures.

**FIGURE 1:**
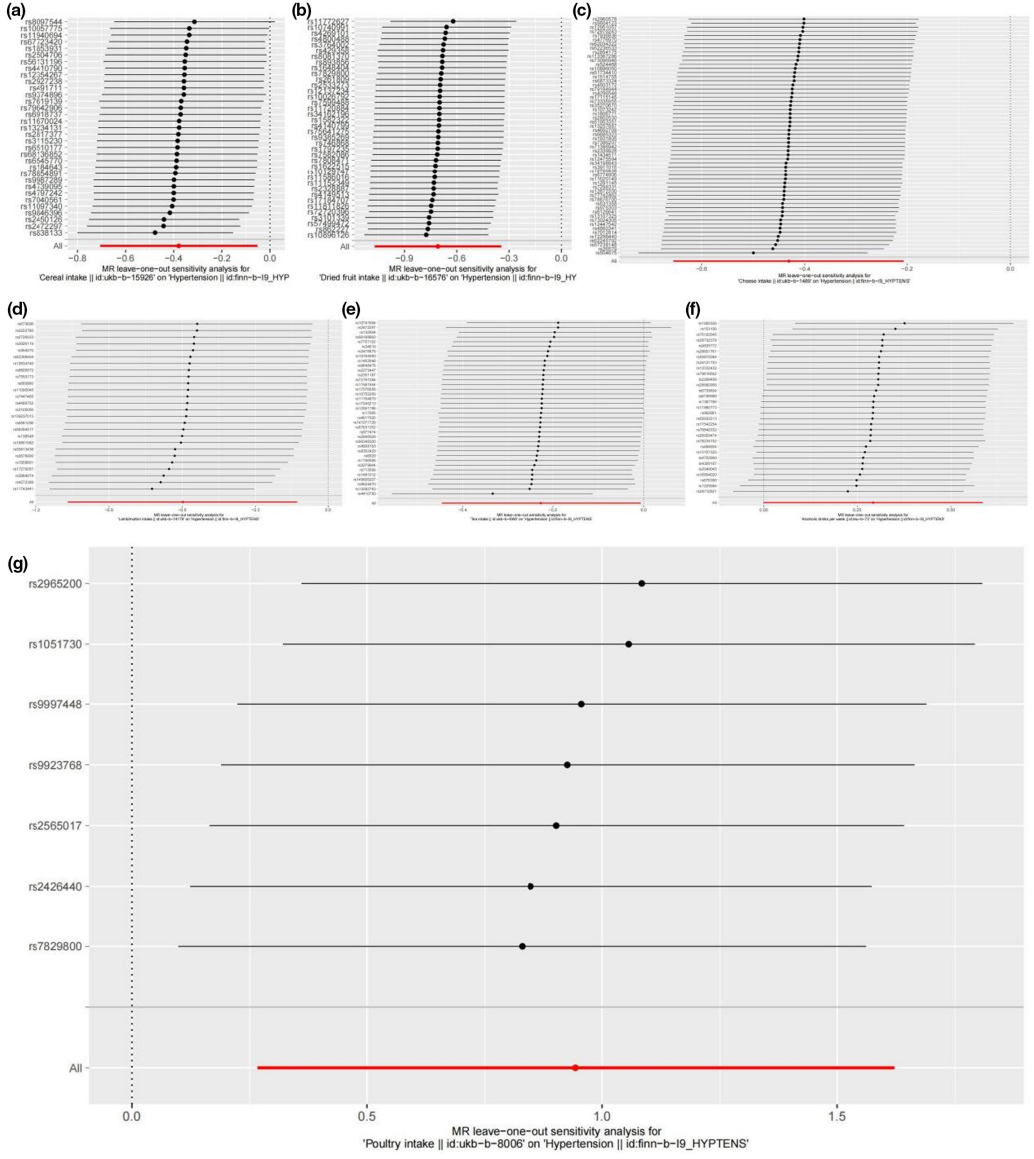
The results of leave‐one‐out analyses. The results of Leave‐one‐out analyses (a) cereal intake, (b) dried fruit intake, (c) cheese intake, (d) lamb/mutton intake, (e) Tea intake, (f) alcoholic drinks per week, (g) poultry intake.

In this study, the IVW identified a total of seven dietary factors that were associated with hypertension. The study found that alcoholic drinks per week (OR 1.340 [95%CI 1.0001–1.794; *p* = .0499]) and poultry consumption (OR 2.569 [95%CI 1.305–5.057; *p* = .00631]) were related to an increased risk of hypertension. The causal relationship between diet and hypertension was further verified using MR‐Egger (alcoholic drinks per week: OR 1.958 [95%CI 1.026–3.737; *p* = .0508]); poultry consumption: (OR 325,129.5 [95%CI 0.0000472–2.239e+14; *p* = .276]); and weighted median (alcoholic drinks per week: OR 1.514 [95%CI 1.044–2.195; *p* = .0288]); poultry: (OR 2.795 [95%CI 1.191–6.562; *p* = .0182]) model. Lamb/mutton (OR 0.550 [95%CI 0.343–0.881; *p* = .0129]), cheese (OR 0.650 [95%CI 0.519–0.813; *p* = .000159]), tea (OR 0.797 [95%CI 0.640–0.993; *p* = .0433]), cereal (OR 0.684 [95%CI 0.494–0.948; *p* = .0227]), and dried fruit (OR 0.492 [95%CI 0.343–0.707; *p* = .000127]) were negatively associated with hypertension. The weighted median model yields similar results (lamb/mutton: OR 0.389 [95%CI 0.219–0.689; *p* = .0012]; cheese: OR 0.673 [95%CI 0.510–0.888; *p* = .00506]; tea consumption: OR 0.776 [95%CI 0.577–1.044; *p* = .0933]; cereal: 0.602 [95%CI 0.4.2–0.903; *p* = .0142]; dried fruit consumption: OR 0.379 [95%CI 0.250–0.575; *p* < .0001]) after removing outliers. Alcohol consumption frequency was causally related to hypertension until abnormal values were removed (*p* = .0259). In addition, fresh fruit (OR 0.972 [95%CI 0.630–1.497; *p* = .896]; outliers excluded: OR 1.124 [95%CI 0.777–1.628; *p* = .535]), beef (OR 0.926 [95%CI 0.303–2.836; *p* = .893]; outliers excluded: OR 1.238 [95%CI 0.593–2.581; *p* = .570]), nonoily fish (OR 1.825 [95%CI 0.609–5.467; *p* = .283]; outliers excluded: OR 1.360 [95%CI 0.730–2.533; *p* = .332]), oily fish (OR 0.854 [95%CI 0.630–1.159; *p* = .312]; outliers excluded: OR 0.793 [95%CI 0.612–1.027; *p* = .0789]), pork (OR 1.948 [95%CI 0.907–4.183; *p* = .0873]), bread consumption (OR 0.775 [95%CI 0.521–1.153; *p* = .208]), cooked vegetable consumption (OR 1.480 [95%CI 0.615–3.561; *p* = .381]; outliers excluded: OR 1.008 [95%CI 0.563–1.803; *p* = .979]), salad/raw vegetable (OR 0.795 [95%CI 0.453–1.397; *p* = .426]), and coffee (OR 1.265 [95%CI 0.873–1.832; *p* = .214]; outliers excluded: OR 1.185 [95%CI 0.847–1.658; *p* = .321]) were not causally related to hypertension (Table 2). Heterogeneity (Q test *p* < .05) was present in numerous dietary factors, but using the MR‐Egger intercept test, the results were not directionally polymorphic (Table 2). The exclusionary analysis indicated that the causality behind the positive findings was highly robust (Figure 1). The effectiveness of the IVW was confirmed using MR‐PRESSO analysis (causal relationships were only shown in alcoholic drinks per week, poultry consumption, lamb/mutton consumption, cheese consumption, tea consumption, cereal consumption, and dried fruit consumption; Table 2). IVs with a strong correlation with dietary factors (*F*: 17.571–116.369) were used in this study (Table 1).

## 4 DISCUSSION

The Mendelian randomization study found that alcoholic drinks per week, poultry consumption, dried fruit consumption, lamb/mutton consumption, cheese consumption, and tea consumption contributed to the risk of hypertension. We would exclude outliers before the research analysis. Other factors (oily fish consumption, alcohol consumption frequency, fresh fruit consumption, nonoily fish consumption, pork consumption, bread consumption, cooked vegetable consumption, beef consumption, salad/raw vegetable consumption, and coffee consumption) showed no significant correlation with the emergence of hypertension. There was a large body of previous research describing the relationship between diet and the development of hypertension. Dietary plans such as the DASH and Mediterranean diets emphasize increased consumption of vegetables and fruits, a low‐sodium diet, and reduced fat intake to lower the risk of hypertension (Ahmed et al., 2020; Theodoridis et al., 2023). Hypertension affects 40% of the world's population and is an important risk for cardiovascular and cerebral disease in humans. People at risk often prevented and controlled hypertension in the first place by modifying their diet, which was also a long‐term approach to nonpharmacological treatment/prevention, and even after increasing medication the same needed to maintain the previous diet structure. Controlled hypertension significantly reduces the risk of various diseases (Thomopoulos et al., 2014). Mendelian randomization studies, with the help of instrumental variables, can exclude reversing causality as well as confounding factors. To verify the accuracy of the MR analysis, sensitivity, and multi‐effect analyses were performed. We used exposures and results from different regions to avoid bias. This study can, therefore, provide more authoritative dietary guidelines and provide reasonable and implementable clinical advice for people with hypertension.

Alcohol's impact on the cardiovascular system is multifaceted and dose dependent. It can contribute to an increase in oxygen free radicals and oxidative stress. Additionally, the metabolites of ethanol can influence the altered activation of the neurohormonal system, specifically the renin–angiotensin–aldosterone system (Roerecke, 2021). Alcohol consumption is a contributing factor to the worldwide burden of disease, injury, and economic costs. The detrimental effects of alcohol use are universal and have a global impact (Kalinowski & Humphreys, 2016). Two recent meta analyses summarize the association between alcohol and the incidence of hypertension (Jung et al., 2020; Liu et al., 2020). Elevated blood pressure increases the risk of cardiovascular disease and people at risk should pay extra attention to blood pressure control (Lewington et al., 2002). Studies show a clear causal relationship between alcohol consumption and hypertension and a clinically significant dose–systolic blood pressure relationship (Roerecke et al., 2017). Alcohol consumption prolongs the QT interval (Kino et al., 1981), has both short‐term and long‐term effects on hypertension (Fernández‐Solà, 2015), and heavy drinkers are often accompanied by other poor lifestyle habits (tobacco; Chan & Anderson, 2014). It has been proposed that moderate alcohol intake may offer some protective benefits for women, whereas in men, there appears to be a linear relationship between the amount of alcohol consumed and blood pressure levels, irrespective of the quantity of alcohol intake (Taylor et al., 2009). If poor health is the reason for reducing the frequency of alcohol consumption, then this would make moderate alcohol consumption appear healthier (less symptomatic of hypertension), which may explain why the frequency of alcohol consumption was not associated with hypertension after excluding outliers in our analysis.

Much cited in previous studies is the DASH diet versus the Mediterranean diet. The Mediterranean diet mainly emphasizes a dietary pattern of grains, fresh fruit and vegetables, legumes, and nuts. This dietary approach also encompasses the inclusion of fish and seafood, low‐fat dairy products, poultry, and eggs. It advocates for a reduction in the consumption of red meat and sweets or sugary beverages while increasing the proportion of fats in the total daily caloric intake (Davis et al., 2015). Study shows a U‐shaped association between poultry consumption, cereal consumption, and new‐onset hypertension (Zhou et al., 2022). The consumption of poultry has been associated with an increased risk of developing hypertension (Zhang & Zhang, 2018). The hypertension risk linked to poultry and red meat intake in our study may relate to Maillard reaction products (MRPs) like heterocyclic amines and advanced glycoxidation end‐products (AGEs) formed during cooking. These compounds enhance oxidative stress and inflammation, potentially impacting hypertension. Moreover, L‐carnitine in red meat, metabolized into trimethylamine‐N‐oxide (TMAO), is associated with cardiovascular risks, including hypertension (Borgi et al., 2015). The grading of evidence levels indicates a potentially negative correlation, suggesting that higher intake of fruits, whole grains, fish, legumes, nuts, and dietary fiber might be correlated with a decreased chance of developing hypertension (Jabbari et al., 2022). This link could be due to the existence of particular bioactive components in these food items. For example, fruits and vegetables are rich in potassium, which helps regulate blood pressure. Whole grains and dietary fiber can improve blood lipid profiles and reduce arterial pressure. Omega‐3 fatty acids, prevalent in fish, are known for their anti‐inflammatory effects and ability to improve endothelial function. Legumes and nuts contain various phytochemicals and antioxidants that contribute to cardiovascular health. These compounds collectively work to modulate blood pressure, thereby reducing hypertension risk (Hosseinpour‐Niazi et al., 2022; Shridhar et al., 2018). A study finds no correlation between cheese consumption and high blood pressure (Hu et al., 2022). A combined higher intake of yogurt along with a higher score on the DASH (“Dietary Approaches to Stop Hypertension”) diet scale has been linked to a decreased occurrence of hypertension (Buendia et al., 2018). Research has suggested that due to its high antioxidant content, coffee consumption may be inversely related to the probability of elevated blood pressure (Ding et al., 2014). The Singapore Chinese Health Study has found that coffee consumption is associated with a reduced risk of developing high blood pressure, but coffee may be responsible for increased risk in daily tea drinkers (Chei et al., 2018). Contrary to some findings, this study did not observe a significant association between coffee consumption and the risk of hypertension. However, a study by Chieng & Kistler (2022) indicated that coffee intake may lower the risk of cardiovascular diseases, and consuming tea could potentially help in preventing metabolic syndrome, this is consistent with the risk of hypertension being reduced by tea drinking in this study. A separate study focusing on elderly Chinese individuals who have lived long lives revealed that daily consumption of green tea increased the risk of high blood pressure by approximately 38% in men, while it showed no significant effect in women (Peng et al., 2021). Currently, there is a scarcity of research investigating the relationship between the consumption of mutton or lamb and the development of hypertension.

Diet may indirectly influence the development of hypertension through the gut microflora and the level of hormones in the blood. Hypertension may develop from ectopic fat stores (Montani et al., 2004), and sodium in food can directly increase systolic blood pressure (He et al., 2020). Maintaining normal blood pressure through a regular diet is a virtuous habit for today's generation, and controlling blood pressure levels is not just for people with hypertension (Lackland & Egan, 2007). Whereas past RCT studies have tended to study hypertension separately from diet and alcohol consumption to avoid interaction, Mendelian randomization trials are not so troubled.

The following limitations exist in this study: Long‐term eating habits rather than short periods could affect the risk of hypertension. There is also variation in the impact of different ethnic areas, and the combined effect of multiple factors may also lead to biased findings. Mendelian randomization is limited in its ability to analyze causality across different ages and sexes, indicating potential complexity in the mechanisms linking exposure and outcome. The *F* statistic, which assesses the correlation between intermediate variables and exposure factors, could potentially compromise the study's accuracy if it registers below 100. Moreover, this study falls short in offering a detailed examination of diverse diet mixes or a thorough comparative analysis of various diet plans. It is also crucial to recognize that hypertension is influenced by a multitude of factors, including physical activity, body composition, and genetics. Future studies are anticipated to delve deeper into multimodal predictive models for hypertension, incorporating these varied factors.

## 5 CONCLUSION

This research revealed associations between the consumption of lamb/mutton, cheese, tea, cereals, and dried fruits with a lowered incidence of hypertension, and the consumption of alcoholic beverages on a weekly basis and the intake of poultry have been linked to the onset of hypertension. At the same time, alcohol consumption frequency, oily fish consumption, fresh fruit consumption, nonoily fish consumption, pork consumption, bread consumption, cooked vegetable consumption, beef consumption, coffee consumption, and salad/raw vegetable consumption were not associated with hypertension.

## AUTHOR CONTRIBUTIONS

**Fanfan Li:** Conceptualization (equal); methodology (equal); writing – original draft (equal); writing – review and editing (equal). **Wenwen Yang:** Data curation (lead); investigation (lead); resources (lead); supervision (lead). **Shuo Sun:** Methodology (equal); validation (equal); writing – original draft (equal). **Wenhui He:** Formal analysis (equal); writing – original draft (equal). **Shangqing Xu:** Formal analysis (equal); visualization (equal). **Biao Han:** Validation (supporting); writing – review and editing (equal). **Minjie Ma:** Funding acquisition (equal); methodology (equal); supervision (equal).

## FUNDING INFORMATION

This study was supported by the Natural Science Foundation of Gansu Province (23JRRA1597).

## CONFLICT OF INTEREST STATEMENT

The authors declare none.

## Supporting information


Table S1.

## Data Availability

All the GWAS data employed in this research can be accessed through the IEU Open GWAS project, which is available at [https://gwas.mrcieu.ac.uk/].

## References

[fsn33931-bib-0001] Ahmed, F. S. , Wade, A. T. , Guenther, B. A. , Murphy, K. J. , & Elias, M. F. (2020). Adherence to a Mediterranean diet associated with lower blood pressure in a US sample: Findings from the Maine‐Syracuse longitudinal study. Journal of Clinical Hypertension (Greenwich, Conn.), 22, 2276–2284. 33045144 10.1111/jch.14068 PMC8029719

[fsn33931-bib-0002] Borgi, L. , Curhan, G. C. , Willett, W. C. , Hu, F. B. , Satija, A. , & Forman, J. P. (2015). Long‐term intake of animal flesh and risk of developing hypertension in three prospective cohort studies. Journal of Hypertension, 33, 2231–2238. 26237562 10.1097/HJH.0000000000000722 PMC4797063

[fsn33931-bib-0003] Borgi, L. , Muraki, I. , Satija, A. , Willett, W. C. , Rimm, E. B. , & Forman, J. P. (2016). Fruit and vegetable consumption and the incidence of hypertension in three prospective cohort studies. Hypertension, 67, 288–293. 26644239 10.1161/HYPERTENSIONAHA.115.06497 PMC5350612

[fsn33931-bib-0004] Buendia, J. R. , Li, Y. , Hu, F. B. , Cabral, H. J. , Bradlee, M. L. , Quatromoni, P. A. , Singer, M. R. , Curhan, G. C. , & Moore, L. L. (2018). Long‐term yogurt consumption and risk of incident hypertension in adults. Journal of Hypertension, 36, 1671–1679. 29952852 10.1097/HJH.0000000000001737 PMC6613217

[fsn33931-bib-0005] Chan, L. N. , & Anderson, G. D. (2014). Pharmacokinetic and pharmacodynamic drug interactions with ethanol (alcohol). Clinical Pharmacokinetics, 53, 1115–1136. 25267448 10.1007/s40262-014-0190-x

[fsn33931-bib-0006] Chei, C. L. , Loh, J. K. , Soh, A. , Yuan, J. M. , & Koh, W. P. (2018). Coffee, tea, caffeine, and risk of hypertension: The Singapore Chinese health study. European Journal of Nutrition, 57, 1333–1342. 28251341 10.1007/s00394-017-1412-4

[fsn33931-bib-0007] Chieng, D. , & Kistler, P. M. (2022). Coffee and tea on cardiovascular disease (CVD) prevention. Trends in Cardiovascular Medicine, 32, 399–405. 34384881 10.1016/j.tcm.2021.08.004

[fsn33931-bib-0008] Davis, C. , Bryan, J. , Hodgson, J. , & Murphy, K. (2015). Definition of the Mediterranean diet; a literature review. Nutrients, 7, 9139–9153. 26556369 10.3390/nu7115459 PMC4663587

[fsn33931-bib-0009] Ding, M. , Bhupathiraju, S. N. , Satija, A. , van Dam, R. M. , & Hu, F. B. (2014). Long‐term coffee consumption and risk of cardiovascular disease: A systematic review and a dose‐response meta‐analysis of prospective cohort studies. Circulation, 129, 643–659. 24201300 10.1161/CIRCULATIONAHA.113.005925 PMC3945962

[fsn33931-bib-0010] Fernández‐Solà, J. (2015). Cardiovascular risks and benefits of moderate and heavy alcohol consumption. Nature Reviews. Cardiology, 12, 576–587. 26099843 10.1038/nrcardio.2015.91

[fsn33931-bib-0011] Filippou, C. , Tatakis, F. , Polyzos, D. , Manta, E. , Thomopoulos, C. , Nihoyannopoulos, P. , Tousoulis, D. , & Tsioufis, K. (2022). Overview of salt restriction in the dietary approaches to stop hypertension (DASH) and the Mediterranean diet for blood pressure reduction. Reviews in Cardiovascular Medicine, 23, 36. 35092228 10.31083/j.rcm2301036

[fsn33931-bib-0012] Guo, Y. , Wang, X. , Jia, P. , You, Y. , Cheng, Y. , Deng, H. , Luo, S. , & Huang, B. (2020). Ketogenic diet aggravates hypertension via NF‐κB‐mediated endothelial dysfunction in spontaneously hypertensive rats. Life Sciences, 258, 118124. 32702443 10.1016/j.lfs.2020.118124

[fsn33931-bib-0013] Hartwig, F. P. , Davey Smith, G. , & Bowden, J. (2017). Robust inference in summary data mendelian randomization via the zero modal pleiotropy assumption. International Journal of Epidemiology, 46, 1985–1998. 29040600 10.1093/ije/dyx102 PMC5837715

[fsn33931-bib-0014] He, F. J. , Tan, M. , Ma, Y. , & MacGregor, G. A. (2020). Salt reduction to prevent hypertension and cardiovascular disease: JACC state‐of‐the‐art review. Journal of the American College of Cardiology, 75, 632–647. 32057379 10.1016/j.jacc.2019.11.055

[fsn33931-bib-0015] Hemani, G. , Zheng, J. , Elsworth, B. , Wade, K. H. , Haberland, V. , Baird, D. , Laurin, C. , Burgess, S. , Bowden, J. , Langdon, R. , Tan, V. Y. , Yarmolinsky, J. , Shihab, H. A. , Timpson, N. J. , Evans, D. M. , Relton, C. , Martin, R. M. , Davey Smith, G. , Gaunt, T. R. , & Haycock, P. C. (2018). The MR‐base platform supports systematic causal inference across the human phenome. eLife, 7, e34408. 29846171 10.7554/eLife.34408 PMC5976434

[fsn33931-bib-0016] Hosseinpour‐Niazi, S. , Hadaegh, F. , Mirmiran, P. , Daneshpour, M. S. , Mahdavi, M. , & Azizi, F. (2022). Effect of legumes in energy reduced dietary approaches to stop hypertension (DASH) diet on blood pressure among overweight and obese type 2 diabetic patients: A randomized controlled trial. Diabetology and Metabolic Syndrome, 14, 72. 35562742 10.1186/s13098-022-00841-w PMC9107125

[fsn33931-bib-0017] Hu, M. J. , Tan, J. S. , Gao, X. J. , Yang, J. G. , & Yang, Y. J. (2022). Effect of cheese intake on cardiovascular diseases and cardiovascular biomarkers. Nutrients, 14, 2936. 35889893 10.3390/nu14142936 PMC9318947

[fsn33931-bib-0018] Jabbari, M. , Eini‐Zinab, H. , Safaei, E. , Poursoleiman, F. , Amini, B. , Babashahi, M. , Barati, M. , & Hekmatdoost, A. (2022). Determination of the level of evidence for the association between different food groups/items and dietary fiber intake and the risk of cardiovascular diseases and hypertension: An umbrella review. Nutrition Research (New York, N.Y.), 111, 1–13. 36780863 10.1016/j.nutres.2022.12.011

[fsn33931-bib-0019] Jung, M. H. , Shin, E. S. , Ihm, S. H. , Jung, J. G. , Lee, H. Y. , & Kim, C. H. (2020). The effect of alcohol dose on the development of hypertension in Asian and Western men: Systematic review and meta‐analysis. The Korean Journal of Internal Medicine, 35, 906–916. 31795024 10.3904/kjim.2019.016 PMC7373951

[fsn33931-bib-0020] Kalinowski, A. , & Humphreys, K. (2016). Governmental standard drink definitions and low‐risk alcohol consumption guidelines in 37 countries. Addiction (Abingdon, England), 111, 1293–1298. 27073140 10.1111/add.13341

[fsn33931-bib-0021] Kino, M. , Imamitchi, H. , Morigutchi, M. , Kawamura, K. , & Takatsu, T. (1981). Cardiovascular status in asymptomatic alcoholics, with reference to the level of ethanol consumption. British Heart Journal, 46, 545–551. 7317220 10.1136/hrt.46.5.545 PMC482694

[fsn33931-bib-0022] Kitt, J. , Fox, R. , Tucker, K. L. , & McManus, R. J. (2019). New approaches in hypertension management: A review of current and developing technologies and their potential impact on hypertension care. Current Hypertension Reports, 21, 44. 31025117 10.1007/s11906-019-0949-4 PMC6483962

[fsn33931-bib-0023] Lackland, D. T. , & Egan, B. M. (2007). Dietary salt restriction and blood pressure in clinical trials. Current Hypertension Reports, 9, 314–319. 17686383 10.1007/s11906-007-0057-8

[fsn33931-bib-0024] Lewington, S. , Clarke, R. , Qizilbash, N. , Peto, R. , Collins, R. , & Prospective Studies Collaboration . (2002). Age‐specific relevance of usual blood pressure to vascular mortality: A meta‐analysis of individual data for one million adults in 61 prospective studies. Lancet (London, England), 360, 1903–1913. 12493255 10.1016/s0140-6736(02)11911-8

[fsn33931-bib-0025] Liu, F. , Liu, Y. , Sun, X. , Yin, Z. , Li, H. , Deng, K. , Zhao, Y. , Wang, B. , Ren, Y. , Liu, X. , Zhang, D. , Chen, X. , Cheng, C. , Liu, L. , Liu, D. , Chen, G. , Hong, S. , Wang, C. , Zhang, M. , & Hu, D. (2020). Race‐ and sex‐specific association between alcohol consumption and hypertension in 22 cohort studies: A systematic review and meta‐analysis. Nutrition, Metabolism, and Cardiovascular Diseases, 30, 1249–1259. 10.1016/j.numecd.2020.03.018 32446870

[fsn33931-bib-0026] Montani, J. P. , Carroll, J. F. , Dwyer, T. M. , Antic, V. , Yang, Z. , & Dulloo, A. G. (2004). Ectopic fat storage in heart, blood vessels and kidneys in the pathogenesis of cardiovascular diseases. International Journal of Obesity and Related Metabolic Disorders, 28(Suppl 4), S58–S65. 15592488 10.1038/sj.ijo.0802858

[fsn33931-bib-0027] Peng, X. , Zhang, M. , Wang, X. , Wu, K. , Li, Y. , Li, L. , Yang, J. , Ruan, Y. , Bai, R. , Ma, C. , & Liu, N. (2021). Sex differences in the association between green tea consumption and hypertension in elderly Chinese adults. BMC Geriatrics, 21, 486. 34493228 10.1186/s12877-021-02431-3 PMC8424953

[fsn33931-bib-0028] Roerecke, M. (2021). Alcohol's impact on the cardiovascular system. Nutrients, 13, 3419. 34684419 10.3390/nu13103419 PMC8540436

[fsn33931-bib-0029] Roerecke, M. , Kaczorowski, J. , Tobe, S. W. , Gmel, G. , Hasan, O. S. M. , & Rehm, J. (2017). The effect of a reduction in alcohol consumption on blood pressure: A systematic review and meta‐analysis. The Lancet. Public Health, 2, e108–e120. 29253389 10.1016/S2468-2667(17)30003-8 PMC6118407

[fsn33931-bib-0030] Sacks, F. M. , Svetkey, L. P. , Vollmer, W. M. , Appel, L. J. , Bray, G. A. , Harsha, D. , Obarzanek, E. , Conlin, P. R. , Miller, E. R. , Simons‐Morton, D. G. , Karanja, N. , Lin, P. H. , Aickin, M. , Most‐Windhauser, M. M. , Moore, T. J. , Proschan, M. A. , & Cutler, J. A. (2001). Effects on blood pressure of reduced dietary sodium and the dietary approaches to stop hypertension (DASH) diet. DASH‐sodium collaborative research group. The New England Journal of Medicine, 344, 3–10. 11136953 10.1056/NEJM200101043440101

[fsn33931-bib-0031] Saklayen, M. G. (2018). The global epidemic of the metabolic syndrome. Current Hypertension Reports, 20, 12. 29480368 10.1007/s11906-018-0812-z PMC5866840

[fsn33931-bib-0032] Shridhar, K. , Satija, A. , Dhillon, P. K. , Agrawal, S. , Gupta, R. , Bowen, L. , Kinra, S. , Bharathi, A. V. , Prabhakaran, D. , Srinath Reddy, K. , Ebrahim, S. , & Indian Migration Study Group . (2018). Association between empirically derived dietary patterns with blood lipids, fasting blood glucose and blood pressure in adults ‐ the India migration study. Nutrition Journal, 17, 15. 29422041 10.1186/s12937-018-0327-0 PMC5806276

[fsn33931-bib-0033] Staiger, D. O. , & Stock, J. H. (1994). Instrumental variables regression with weak instruments. National Bureau of Economic Research.

[fsn33931-bib-0034] Sudlow, C. , Gallacher, J. , Allen, N. , Beral, V. , Burton, P. , Danesh, J. , Downey, P. , Elliott, P. , Green, J. , Landray, M. , Liu, B. , Matthews, P. , Ong, G. , Pell, J. , Silman, A. , Young, A. , Sprosen, T. , Peakman, T. , & Collins, R. (2015). UK biobank: An open access resource for identifying the causes of a wide range of complex diseases of middle and old age. PLoS Medicine, 12, e1001779. 25826379 10.1371/journal.pmed.1001779 PMC4380465

[fsn33931-bib-0035] Taskinen, M. R. , Packard, C. J. , & Borén, J. (2019). Dietary fructose and the metabolic syndrome. Nutrients, 11, 1987. 31443567 10.3390/nu11091987 PMC6770027

[fsn33931-bib-0036] Taylor, B. , Irving, H. M. , Baliunas, D. , Roerecke, M. , Patra, J. , Mohapatra, S. , & Rehm, J. (2009). Alcohol and hypertension: Gender differences in dose‐response relationships determined through systematic review and meta‐analysis. Addiction (Abingdon, England), 104, 1981–1990. 19804464 10.1111/j.1360-0443.2009.02694.x

[fsn33931-bib-0037] Theodoridis, X. , Chourdakis, M. , Chrysoula, L. , Chroni, V. , Tirodimos, I. , Dipla, K. , Gkaliagkousi, E. , & Triantafyllou, A. (2023). Adherence to the DASH diet and risk of hypertension: A systematic review and meta‐analysis. Nutrients, 15, 3261. 37513679 10.3390/nu15143261 PMC10383418

[fsn33931-bib-0038] Thomopoulos, C. , Parati, G. , & Zanchetti, A. (2014). Effects of blood pressure lowering on outcome incidence in hypertension: 3. Effects in patients at different levels of cardiovascular risk–overview and meta‐analyses of randomized trials. Journal of Hypertension, 32, 2305–2314. 25259548 10.1097/HJH.0000000000000380

[fsn33931-bib-0039] Tseng, E. , Appel, L. J. , Yeh, H. C. , Pilla, S. J. , Miller, E. R. , Juraschek, S. P. , & Maruthur, N. M. (2021). Effects of the dietary approaches to stop hypertension diet and sodium reduction on blood pressure in persons with diabetes. Hypertension, 77, 265–274. 33342238 10.1161/HYPERTENSIONAHA.120.14584 PMC7810162

[fsn33931-bib-0040] Volek, J. S. , Phinney, S. D. , Forsythe, C. E. , Quann, E. E. , Wood, R. J. , Puglisi, M. J. , Kraemer, W. J. , Bibus, D. M. , Fernandez, M. L. , & Feinman, R. D. (2009). Carbohydrate restriction has a more favorable impact on the metabolic syndrome than a low fat diet. Lipids, 44, 297–309. 19082851 10.1007/s11745-008-3274-2

[fsn33931-bib-0041] Williams, B. , Mancia, G. , Spiering, W. , Agabiti Rosei, E. , Azizi, M. , Burnier, M. , Clement, D. , Coca, A. , de Simone, G. , Dominiczak, A. , Kahan, T. , Mahfoud, F. , Redon, J. , Ruilope, L. , Zanchetti, A. , Kerins, M. , Kjeldsen, S. , Kreutz, R. , Laurent, S. , … *Task Force members* . (2018a). 2018 practice guidelines for the management of arterial hypertension of the European Society of Hypertension and the European Society of Cardiology: ESH/ESC task force for the Management of Arterial Hypertension. Journal of Hypertension, 36, 2284–2309. 30379783 10.1097/HJH.0000000000001961

[fsn33931-bib-0042] Williams, B. , Mancia, G. , Spiering, W. , Agabiti Rosei, E. , Azizi, M. , Burnier, M. , Clement, D. L. , Coca, A. , de Simone, G. , Dominiczak, A. , Kahan, T. , Mahfoud, F. , Redon, J. , Ruilope, L. , Zanchetti, A. , Kerins, M. , Kjeldsen, S. E. , Kreutz, R. , Laurent, S. , … *ESC Scientific Document Group* . (2018b). 2018 ESC/ESH guidelines for the management of arterial hypertension. European Heart Journal, 39, 3021–3104. 30165516 10.1093/eurheartj/ehy339

[fsn33931-bib-0043] Zhang, Y. , & Zhang, D. Z. (2018). Red meat, poultry, and egg consumption with the risk of hypertension: A meta‐analysis of prospective cohort studies. Journal of Human Hypertension, 32, 507–517. 29725070 10.1038/s41371-018-0068-8

[fsn33931-bib-0044] Zhou, B. , Perel, P. , Mensah, G. A. , & Ezzati, M. (2021). Global epidemiology, health burden and effective interventions for elevated blood pressure and hypertension. Nature Reviews. Cardiology, 18, 785–802. 34050340 10.1038/s41569-021-00559-8 PMC8162166

[fsn33931-bib-0045] Zhou, C. , Wu, Q. , Ye, Z. , Liu, M. , Zhang, Z. , Zhang, Y. , Li, H. , He, P. , Li, Q. , Liu, C. , & Qin, X. (2022). Inverse association between variety of proteins with appropriate quantity from different food sources and new‐onset. Hypertension Hypertension (Dallas, Tex.: 1979), 79, 1017–1027. 35264000 10.1161/HYPERTENSIONAHA.121.18222

